# Train-your-brain pilot community-based intervention after stroke: cognitive trajectory over 10-month follow-up

**DOI:** 10.3389/fneur.2025.1500943

**Published:** 2025-07-07

**Authors:** Nicole Yun Ching Chen, Xiang Cong Tham, Yanhong Dong

**Affiliations:** ^1^Yong Loo Lin School of Medicine, National University of Singapore, Singapore, Singapore; ^2^Alice Lee Centre for Nursing Studies, National University of Singapore, Singapore, Singapore

**Keywords:** stroke survivors, caregivers, community intervention, cognition, cognitive functioning

## Abstract

**Introduction:**

Stroke leads to cognitive impairments that affect survivors’ quality of life. This study aimed to assess the effectiveness of the Train-Your-Brain (TYB) pilot community intervention in cognitive outcomes among stroke survivors and caregivers at baseline, post-intervention, and 10-month follow-up.

**Methods:**

Thirty-one participants (20 stroke survivors, 11 caregivers) were evaluated. Cognitive functioning was measured using the Montreal Cognitive Assessment (MoCA) with analysis of subtest-level performances and Symbol Digit Modalities Test (SDMT).

**Results:**

Among stroke survivors, MoCA immediate recall scores maintained during the intervention, but declined 10-months later (*p* = 0.005). Analysis of the MoCA delayed memory subtest revealed a graded performance across different recall formats. Free recall and category-cued recall deteriorated over 10 months, while multiple-choice format recall remained stable. A slight improvement was observed in SDMT scores from pre-TYB to post-TYB, which was relatively maintained after 10 months. Caregivers demonstrated significant improvements in MoCA language and sentence repetition (*p* = 0.014) scores at 10-month follow-up.

**Conclusion:**

Our findings suggest that while the intervention may lead to short-term stabilization in cognitive functioning among stroke survivors, these gains may not be sustained over time. Persistent cognitive deficits underscore the need for ongoing and long-term support.

## 1 Introduction

Stroke is Singapore’s fourth leading cause of death and the seventh leading cause of adult disability (1). It is a major public health burden in Asia (2) and expected to rise with Singapore’s aging population. The aftermath of a stroke brings forth significant changes to one’s functioning and quality of life, with survivors commonly experiencing vascular cognitive impairment (3).

Vascular cognitive impairment refers to cognitive impairment related to vascular diseases such as stroke, leading to increased subcortical white matter hyperintensities in the brain, and a neuropsychological profile reflecting frontosubcortical deficits (4). Such deficits are characterized by impairments in processing speed and executive functioning, although presentations can vary depending on factors such as stroke type, lesion location, and implicated regions in the brain (5, 6). Stroke survivors commonly experience challenges associated with aphasia, apraxia, and hemispatial neglect (7). A systematic review and meta-analysis across 16 hospital-based studies revealed that 53.4% of stroke survivors experienced post-stroke cognitive impairment (8). While recovery can occur especially in the acute periods post-stroke, up to one-third of stroke survivors develop dementia within 5 years (9). Cognitive impairment significantly impacts survivors’ quality of life (10), interferes with their social engagement and rehabilitation.

International consensus-based core recommendations in stroke rehabilitation and recovery identified cognitive function as an area of unmet need, demonstrating the importance of post-stroke cognitive interventions (11). Current cognitive interventions typically teach restorative (i.e., restoring lost skills) or compensatory approaches (i.e., teaching compensatory techniques) (10), however it is still uncertain as to which is the best treatment approach to remediate cognitive impairment. A systematic review and meta-analysis evaluating 22 randomized controlled trials of cognitive remediation post-stroke revealed that cognitive remediation interventions have a positive impact on cognitive outcomes, with stroke survivors demonstrating improvement in cognitive functioning of a small overall effect (*g* = 0.48) compared to control groups (12). Furthermore, a small overall effect (*g* = 0.27) was sustained at follow-up ranging from 2 to 52 weeks. However, a review of Cochrane systematic reviews and randomized controlled trials for rehabilitation trials for post-stroke cognitive impairment reported favorable outcomes in attention, spatial neglect, and motor apraxia immediately post-intervention, though these improvements have been not sustained at the long-term follow-up (10). There has been insufficient research evidence to support the effectiveness of cognitive rehabilitation after stroke, and thus a need to strengthen the evidence base (10).

Stroke services in Singapore typically target physical and daily functioning outcomes, with little focus on cognitive rehabilitation or psychological wellbeing. Stroke survivors generally receive outpatient rehabilitation services upon discharge, however the compliance rate has been low, due to factors including mobility difficulties, lack of transportation access, financial constraints, and cognitive impairment which limit successful engagement in rehabilitation (13). Recognizing the multifaceted nature of the post-stroke recovery process, the Train-Your-Brain (TYB) community-based intervention was developed locally to address the unique needs of both stroke survivors and their caregivers (14). It is conducted virtually to eliminate barriers such as mobility and access difficulties. The intervention content included psychoeducation on poststroke recovery, lifestyle interventions, cognitive training (including memory, attention, and executive functioning strategies), and management of post-stroke mood difficulties and fatigue.

The current study aimed to assess the effectiveness of the pilot TYB intervention in cognitive outcome and its trajectory of stroke survivors and caregivers at baseline, upon intervention completion, and at 10-months follow-up. By including caregivers, the study also aimed to explore the broader impact of the intervention on caregivers who play a pivotal role in the recovery process of stroke survivors. The findings have the potential to guide future rehabilitation studies for post-stroke cognitive impairment, contributing to the development of evidence-based and scalable interventions tailored to address the complex and persistent cognitive challenges faced by stroke survivors.

## 2 Methods

### 2.1 Study design

This study adopted a quasi-experimental design. Sixty-four participants were initially recruited from a community-based brain health intervention for stroke recovery (14). Recruitment was carried out from day rehabilitation centers, primary healthcare polyclinics, stroke associations, and word of mouth. Among them, 56 participants consented to participate. Inclusion criteria were either: (1) stroke survivors who were at least 3 months post-stroke and who did not have significant aphasia or apraxia that would prevent them from providing informed consent, or (2) any caregivers of stroke survivors. All participants were fluent in English. This study obtained institutional ethics approval from the National University of Singapore-Institutional Review Board (NUS-IRB-2021-412).

### 2.2 Intervention

As previously described (14), 48 participants (27 stroke survivors and 21 caregivers) completed the TYB intervention between October and December 2022. The intervention was delivered over nine sessions over 2 months. Sessions were conducted once to twice a week, with a duration of 60–90 min per session. The group-based sessions were delivered by experienced clinical neuropsychologists online via the Zoom video conferencing software application.

The intervention content included: memory and thinking difficulties related to stroke; impact of health and lifestyle on cognition and stroke recovery; managing mood to improve cognitive functioning; improving attention and memory; learning good planning and organization skills to optimize recovery; psychoeducation on return to work after stroke; managing fatigue, mood, and stress at work; applying memory and executive functioning strategies to work effectively; and concluding with a review session of key takeaways from the intervention. At the start of each session, participants were provided with a concise recap of the previous session to reinforce key concepts. Participants were encouraged to share their personal experiences and thoughts during the session. They were also assigned homework at the end of each session to reinforce their learning. No additional sessions were held after the intervention concluded. Table 1 describes the TYB intervention outline. Details on the intervention program can be obtained from Tham et al. (14).

**Table 1:** TYB intervention outline with session content and activities.

Session	Session focus	Content/activities
1	Memory and thinking difficulties related to stroke and recovery	Setting ground rules and expectations Overview of stroke processes and common cognitive difficulties after stroke
2	Lifestyle factors to prevent the recurrence of stroke	Common risk factors of stroke Lifestyle changes to improve brain health
3	Mood and cognitive functioning	Effect of mood on memory and cognition Mood and stress management tools and exercises
4	Attention and memory	Types of attention and its impact to memory Practical strategies to improve attention and focus
6	Executive functioning	Introduction to executive functioning skills Practical strategies to improve executive functioning (e.g., organization tips, SMART goal setting)
7	Review of strategies	Review key points from previous sessions Discuss compensatory strategies and memory aids Introduction to cognitive stimulation activities
8	When to return to work	Understanding recovery and return to work capacity Goal setting for return to work
9	Managing fatigue and mood at work	Identification of post-stroke fatigue and coping strategies Identification of work stressors and stress management tips
10	Applying memory and thinking strategies to work effectively	Review of cognitive strategies Application of cognitive strategies to work environment
11	Booster Session	Review practice and progress Program graduation ceremony

### 2.3 Measurements

Before the commencement of the intervention, trained research team members conducted baseline assessments during home visits. After completion of the intervention, research team members visited participants’ homes for post-intervention assessments. During the home visits, demographic characteristics were obtained and the participants were screened for their cognition and mood. At 10-months after the intervention completion, 31 participants were visited for a follow-up assessment, where they received the Montreal Cognitive Assessment (MoCA) and Symbol Digit Modalities Test (SDMT).

The MoCA (15) and SDMT (16) were administered to evaluate cognitive functioning. The MoCA is a brief cognitive screening tool comprised of subtests examining cognitive domains of visuospatial/executive functioning, naming, memory, attention, language, abstraction, delayed recall, and orientation. Each cognitive domain consisting of 1 or more subtests is given a sub-score, contributing to a total score of 30 points. In this study, a modified version of MoCA tailored to the Singaporean population was used; its validation has been described previously (17, 18). The SDMT is a visuomotor processing speed test that has been widely used and validated in stroke populations, with significant correlation with stroke severity (19–21). The inclusion of the SDMT alongside the MoCA was prompted by the absence of a processing speed measure in the MoCA, a domain which is frequently impaired in stroke patients (6, 22). A lower score on both the MoCA and SDMT indicates greater severity of cognitive impairment. Research has established that when utilized along with the MoCA, the SDMT enhances accuracy in screening for cognitive impairment in vascular diseases (20).

### 2.4 Statistical analysis

Normality was not assumed given the small sample size (*n* = 31). Mann–Whitney U-test was conducted to compare outcomes between the groups (stroke survivors and caregivers). A two-tailed value of p of less than 0.05 was considered as statistically significant. Friedman test was conducted to measure changes in cognition amongst participants based on the SDMT, MoCA total, and MoCA subtest domain scores. *Post hoc* analysis with Wilcoxon Signed-Rank tests was conducted with a Bonferroni correction applied, resulting in a significance level set at *p* < 0.017. Spearman’s rank-order correlations was performed to explore the relationships between cognitive scores. The analyses were conducted using SPSS Version 29.

## 3 Results

### 3.1 Participant characteristics

Participants were visited for follow-up assessments at 10 months after the completion of the TYB intervention. At this follow-up, 31 participants remained, consisting of 20 stroke survivors and 11 caregivers. Seventeen participants (33.3%) were lost to follow-up due to various reasons—three caregivers were not followed up due to stroke survivor’s demise, six participants were abroad, four participants declined follow-up, and four participants were uncontactable. The flow diagram of participation and reasons for attrition can be found in Supplementary Figure 1. Most of the participants were Chinese (83.9%) males (51.6%). Participants had a median age of 61 ± 19 years, with a median years of education of 15 ± 4 years. Among the stroke survivors, five had ischaemic strokes, 13 had haemorrhagic strokes, one had a transient ischaemic attack, and one was unknown.

### 3.2 Comparison of SDMT and MoCA scores between participant groups

Before participating in the TYB intervention, stroke survivors had significantly lower MoCA scores compared to caregivers (26 ± 4.75 vs. 29 ± 1, *p* = 0.004). Post-TYB, there were no significant differences in MoCA scores between stroke survivors and caregivers (27 ± 3 vs. 28 ± 2, *p* = 0.157). However, at 10-months follow-up, a significant difference in MoCA scores was again found between stroke survivors and caregivers (26 ± 5.5 vs. 29 ± 3, *p* = 0.008). Pre-TYB, stroke survivors exhibited significantly lower SDMT scores compared to caregivers (31.5 ± 20.5 vs. 57 ± 22, *p* < 0.001). This disparity persisted post-TYB (*p* < 0.001), with stroke survivors’ SDMT scores remaining significantly lower than those of caregivers (35.5 ± 16.75 vs. 56 ± 21). Similarly, at 10-months follow-up, stroke survivors continued to demonstrate significantly lower SDMT scores in comparison to caregivers (34 ± 22.5 vs. 56 ± 21, *p* < 0.001).

### 3.3 Analysis of MoCA and SDMT scores in stroke survivors

A statistically significant difference was found in scores on the memory item (2nd trial) among stroke survivors, (*χ*^2^(2) = 12.80, *p* = 0.002) (Table 2). This item measured immediate recall of a list of words. *Post hoc* analysis with Wilcoxon signed-rank tests was conducted with a Bonferroni correction applied, resulting in a significance level set at *p* < 0.017. There was a statistically significant decrease in immediate memory item scores at 10-months follow-up versus pre-TYB (*Z* = −2.828, *p* = 0.005), as well as at 10-month follow-up versus post-TYB (*Z* = −2.828, *p* = 0.005). There were no significant differences between the pre-TYB and post-TYB scores (*Z* = 0.000, *p* = 1.000). Median (IQR) score for memory item at pre-TYB, post-TYB, and 10-months follow-up was 5 (0), 5 (0), and 4.5 (1), respectively.

**Table 2:** Comparison of SDMT and MoCA scores in stroke survivors pre-intervention, post-intervention, and at 10-months follow-up (*n* = 20).

	Pre-TYB (a) (Median ± IQR)	Post-TYB (b) (Median ± IQR)	Follow-up (c) (Median ± IQR)	Asymp. sig	Pairwise comparison
SDMT	31.5 ± 20.5	35.5 ± 16.75	34 ± 22.5	0.302	–
Total MoCA score	26 ± 4.75	27 ± 3	26 ± 5.5	0.418	–
MoCA: Memory					
1st trial	5 ± 1	5 ± 1	4 ± 0.75	0.217	–
2nd trial	5 ± 0	5 ± 0	4.5 ± 1	0.002	a > c*; b > c*
MoCA: Delayed recall					
With no cue	5 ± 1	5 ± 1	4 ± 2	0.027	n.s
With category cue	5 ± 0.75	5 ± 0	4.5 ± 1	0.053	n.s.
With multiple choice	5 ± 0	5 ± 0	5 ± 0	0.819	–
MoCA: Visuospatial/executive	4.5 ± 1	4 ± 1.75	4.5 ± 1	0.458	–
Alternating trail making	1 ± 0.75	1 ± 0	1 ± 0	0.565	–
Cube	1 ± 0.75	0.5 ± 1	1 ± 0.75	0.044	n.s
Clock	3 ± 1	3 ± 1	3 ± 1	0.905	–
MoCA: Naming	3 ± 0	3 ± 0	3 ± 0	0.368	–
Lion	1 ± 0	1 ± 0	1 ± 0	0.368	–
Elephant	1 ± 0	1 ± 0	1 ± 0	–	–
Camel	1 ± 0	1 ± 0	1 ± 0	–	–
MoCA: Attention	6 ± 0.75	6 ± 0.75	6 ± 1	0.328	–
Digit forwards	1 ± 0	1 ± 0	1 ± 0	0.607	–
Digit backwards	1 ± 0	1 ± 0	1 ± 0	0.449	–
Vigilance	1 ± 0	1 ± 0	1 ± 0.75	0.417	–
Serial 7s	3 ± 0	3 ± 0	3 ± 1	0.021	n.s
MoCA: Language	2 ± 1	2 ± 1.75	2 ± 2	0.103	–
Sentence repetition	1 ± 0	1 ± 1.75	1 ± 2	0.274	–
Fluency	1 ± 0	1 ± 0	1 ± 0	0.846	–
MoCA: Abstraction	2 ± 0	2 ± 0	2 ± 0.75	1	–
Train/bicycle	1 ± 0	1 ± 0	1 ± 0	0.135	–
Watch/ruler	1 ± 0	1 ± 0	1 ± 0	0.513	–
MoCA: Orientation	6 ± 1	6 ± 0	6 ± 0.75	0.482	–

IQR = interquartile range.

*Significant pairwise comparisons.

On the MoCA, there were also statistically significant differences in stroke survivors’ scores on the cube item (*χ*^2^(2) = 6.250, *p* = 0.044), Serial 7s item (*χ*^2^(2) = 7.750, *p* = 0.021), and delayed recall item when no cues were provided (*χ*^2^(2) = 7.256, *p* = 0.027). Borderline significant differences were also seen on the delayed recall item when category cues were provided (*χ*^2^(2) = 5.886, *p* = 0.053). However, after conducting post-hoc analysis with Wilcoxon signed-rank tests and applying a Bonferroni correction, these significant differences were not maintained. When multiple choice cues were provided in the delayed recall item, there were no significant differences seen (*p* = 0.819).

Table 2 also reveals an increase in SDMT scores from 31.5 ± 20.5 pre-TYB to 35.5 ± 16.75 post-TYB and 34 ± 22.5 at 10-months follow-up, however *p* value did not indicate statistical significance.

### 3.4 Correlations between MoCA and SDMT scores in stroke survivors

Spearman’s rank-order correlations showed moderate positive correlations between SDMT scores and total MoCA scores at pre-TYB (r_s_(18) = 0.553, *p* = 0.011), post-TYB (r_s_(18) = 0.692, *p* < 0.001), and 10-months follow-up (r_s_(18) = 0.555, *p* = 0.011). These findings suggest that higher SDMT scores which reflect better processing speed, are associated with higher total MoCA scores at all time points.

Examining the MoCA subtest domain scores, there was moderate positive correlation between immediate memory item score and total MoCA score (r_s_(18) = 0.523, *p* = 0.018) at 10-months follow-up, however this correlation was not found pre-TYB and post-TYB. At 10-months follow-up, there was moderate positive correlation between scores of immediate memory with attention (r_s_(18) = 0.556, *p* = 0.011) and delayed recall (r_s_(18) = 0.648, *p* = 0.002), which were statistically significant. This suggests that participants with higher immediate memory scores tended to have higher scores in attention, delayed recall, and overall cognitive functioning at 10-month follow-up.

### 3.5 Analysis of MoCA and SDMT scores in caregivers

There was a statistically significant difference in caregivers’ language and sentence repetition scores on the MoCA, both at *χ*^2^(2) = 6.937, *p* = 0.031 (Table 3). *Post hoc* analysis with Wilcoxon signed-rank tests was conducted with a Bonferroni correction applied, resulting in a significance level set at *p* < 0.017. Median (IQR) score for language at pre-TYB, post-TYB, and 10-month follow-up was 2 (2), 2 (1), and 3 (1), respectively. There was a statistically significant increase in language scores at 10-month follow-up versus post-TYB (*Z* = −2.460, *p* = 0.014). There were no significant differences between the pre-TYB and post-TYB scores (*Z* = −1.414, *p* = 0.157). Median (IQR) score for sentence repetition item at pre-TYB, post-TYB, and 10-month follow-up was 1 (2), 1 (1), and 2 (1), respectively. Similarly, there was a statistically significant increase in sentence repetition scores at 10-month follow-up versus post-TYB (*Z* = −2.460, *p* = 0.014). There were no significant differences between the pre-TYB and post-TYB scores (*Z* = −1.414, *p* = 0.157). The significant increase in language subdomain seems to be driven by the significant increase in sentence repetition scores.

**Table 3:** Comparison of SDMT and MoCA scores in caregivers pre-intervention, post-intervention, and at 10-months follow-up (*n* = 11).

	Pre-TYB (a) (Median ± IQR)	Post-TYB (b) (Median ± IQR)	Follow-up (c) (Median ± IQR)	Asymp. sig	Pairwise comparison
SDMT	57 ± 22	56 ± 21	56 ± 21	0.695	–
Total MoCA score	29 ± 1	28 ± 2	29 ± 3	0.187	–
MoCA: Memory					
1st trial	5 ± 0	5 ± 0	5 ± 1	0.105	–
2nd trial	5 ± 0	5 ± 0	5 ± 0	0.223	–
MoCA: Delayed recall					
With no cue	5 ± 0	5 ± 0	5 ± 1	0.292	–
With category cue	5 ± 0	5 ± 0	5 ± 0	0.368	–
With multiple choice	5 ± 0	5 ± 0	5 ± 0	0.368	–
MoCA: Visuospatial/executive	5 ± 0	4 ± 1	5 ± 1	0.135	–
Alternating trail making	1 ± 0	1 ± 0	1 ± 0	0.607	–
Cube	1 ± 0	1 ± 1	1 ± 0	0.135	–
Clock	3 ± 0	3 ± 1	3 ± 0	0.368	–
MoCA: Naming	3 ± 0	3 ± 0	3 ± 0	–	–
Lion	1 ± 0	1 ± 0	1 ± 0	–	–
Elephant	1 ± 0	1 ± 0	1 ± 0	–	–
Camel	1 ± 0	1 ± 0	1 ± 0	–	–
MoCA: Attention	6 ± 0	6 ± 0	6 ± 0	0.779	–
Digit forwards	1 ± 0	1 ± 0	1 ± 0	–	–
Digit backwards	1 ± 0	1 ± 0	1 ± 0	0.368	–
Vigilance	1 ± 0	1 ± 0	1 ± 0	–	–
Serial 7s	3 ± 0	3 ± 0	3 ± 0	0.717	–
MoCA: Language	2 ± 2	2 ± 1	3 ± 1	0.031	c > b*
Sentence repetition	1 ± 2	1 ± 1	2 ± 1	0.031	c > b*
Fluency	1 ± 0	1 ± 0	1 ± 0	–	–
MoCA: Abstraction	2 ± 0	2 ± 0	2 ± 0	–	–
Train/bicycle	1 ± 0	1 ± 0	1 ± 0	–	–
Watch/ruler	1 ± 0	1 ± 0	1 ± 0	–	–
MoCA: Orientation	6 ± 0	6 ± 0	6 ± 0	0.368	–

IQR = interquartile range.

*Significant pairwise comparisons.

## 4 Discussion

This study was a pilot trial that examined the cognitive outcomes of stroke survivors and caregivers at three time points: pre-intervention, post-intervention, and at 10-months follow-up. We utilized two established cognitive assessment tools, the SDMT and MoCA, to measure these outcomes. While previous research has analyzed the subdomains of the MoCA (i.e., visuospatial/executive functioning, naming, memory, attention, language, abstraction, delayed recall, orientation), this is the first study till date to examine the individual item performances on the MoCA in a longitudinal stroke intervention study. By examining performance at the item level, we are able to pinpoint which cognitive elements are most responsive to intervention, even when utilizing a cognitive screening measure. This study found that the TYB intervention led to short-term stabilization of immediate recall among stroke survivors, however this was not sustained at 10-month follow-up. Analysis of the MoCA delayed memory subtest revealed a graded performance across different recall formats where free recall and category-cued recall deteriorated over 10 months, while multiple-choice format recall remained stable. A slight improvement was observed in SDMT scores from pre-TYB to post-TYB, which was relatively maintained after 10 months. Among caregivers, significant improvements were observed in language and sentence repetition at 10-month follow-up.

Studies show that episodic memory is impaired in 25–46% of stroke survivors (23, 24). Stroke survivors have been found to experience greater difficulties with encoding new information compared to retention of information, which was attributed to frontosubcortical difficulties such as attention, processing speed, and executive functioning (24–26). In assessing memory, tasks mainly evaluate an individual’s encoding and retrieval abilities, utilizing free recall, cued recall, or recognition formats for the retrieval process (27). In the MoCA, the retrieval formats include a free recall, cued recall (categorical relatedness), and recognition (multiple-choice options). In this study, we examined the differences in performance across all retrieval formats. Among stroke survivors, analysis of MoCA subtests revealed that immediate recall scores (measured by the memory item) maintained from pre-TYB to post-TYB, but declined 10-months later. This suggested that while immediate memory (i.e., encoding) appeared to be maintained during the program, this was not sustained in the long term. This may be explained in part by the TYB intervention’s heavier emphasis on teaching memory strategies as well as giving participants homework to train their memory in between sessions.

Executive functioning deficits also contribute to retrieval deficits, while retention is relatively preserved. This shows up in difficulties with spontaneous retrieval but improved performance upon cueing or recognition format. The use of cues, prompts, or context acts as a scaffold, which aids retrieval of information that was previously encoded. The recognition format typically offers the highest level of context and scaffold that aids retrieval processes. In this study, further analysis of the MoCA delayed memory subtest among stroke survivors revealed a graded performance across different recall formats. Free recall and category-cued recall deteriorated over 10 months, while multiple-choice format recall remained stable over time. Participants exhibited better performance in the multiple-choice format, followed by category cueing, then free recall. This profile typically suggests that executive functioning difficulties interfered with memory processes. This finding can further guide targeted rehabilitation strategies for stroke survivors, emphasizing the greater benefit of focusing on encoding and retrieval abilities (e.g., structured memory aids, visual cues, mnemonic strategies) to bolster memory functioning.

Furthermore, participants with higher immediate memory scores tended to demonstrate higher scores in attention, delayed recall, and overall cognitive functioning at 10-month follow-up. Rehabilitation efforts could focus on enhancing attention, potentially improving immediate memory and consequently better delayed recall. Encouragingly, a slight improvement was observed in SDMT scores among stroke survivors from pre-TYB to post-TYB, and these scores were relatively maintained at 10-months follow-up. Furthermore, positive correlations were found between SDMT scores and total MoCA scores at all time points in stroke survivors, highlighting the importance of processing speed in post-stroke cognitive functioning. Stroke survivors with higher immediate memory scores also tended to have higher scores in attention, delayed recall, and overall cognitive functioning at 10-month follow-up. Caregivers demonstrated significant improvements in language and sentence repetition scores on the MoCA at 10-month follow-up when compared to post-TYB, suggesting potential cognitive benefits from the TYB program for caregivers as well.

We anticipated that the cognitive exercises and strategies delivered during the TYB intervention would lead to cognitive improvements, however the findings indicated that while initial stabilization occurred, these gains were not maintained at follow-up. We explore several explanations for the findings obtained. The evidence surrounding cognitive rehabilitation intervention and outcomes following a stroke remain unclear and lacking in robustness, although recent findings are showing some support for such interventions. A recent systematic review and meta-analysis of 64 randomized controlled trials showed that multiple component interventions such as cognitive rehabilitation and physical activity interventions tend to show improvements in memory and overall cognitive functioning, compared to single component interventions (28). Furthermore, domain-specific cognitive rehabilitation have been found to be limiting as cognitive domains are highly overlapping and cognitive impairments tend to be heterogenous post-stroke (28). The TYB intervention’s focus on training specific cognitive domains (i.e., memory, attention, executive functioning) may have limited its benefit on other cognitive domains. Improvements on the intervention can be made by expanding it to target general cognition as well as incorporating other components of recovery.

In their review of cognitive remediation interventions post-stroke, Rogers et al. (12) found that the positive effects were moderated by factors including stage of recovery, study quality, and dose (i.e., duration, frequency). Best practice guidelines recommend commencement of stroke rehabilitation in the early recovery phase post-stroke once patients are medically stable (29). As this was a pilot study, there were no restrictions on duration since stroke. Hence, participants at various stages of stroke recovery, including those in the chronic phase, were included. This heterogeneity in participants’ post-stroke timelines may have affected the cognitive outcomes, as participants in the chronic phase may have already experienced stabilization or plateauing in their cognitive functioning. Research has shown that improvements in cognition generally occurred during the first 3 months and plateaued at 12 months post-stroke, although this varied depending on baseline cognitive status with no improvement found for patients with severe cognitive impairment (30). This plateauing effect could potentially attenuate the potential cognitive gains from the intervention program among these individuals. Moreover, the optimal dose and frequency of post-stroke cognitive interventions has yet to be established in the literature (11, 31), hence this may be an unknown influence on the cognitive outcomes. Incorporating booster sessions, activity monitoring, or feedback systems may be more effective in sustaining long-term treatment gains (12). Furthermore, it is not yet clear if the benefit from cognitive rehabilitation is equal across age ranges, that is whether older stroke survivors have the same potential for cognitive improvements as younger stroke survivors (10). The stroke survivors in this study had a median age of 64, placing them within the older adults age group where aging and neurodegenerative processes are taking place. Therefore, observing a stabilizing effect on cognitive scores (i.e., maintenance of cognitive function) may be interpreted as a favorable outcome in the prevention of cognitive decline and dementia.

Overall, insights gathered from this study suggest that future studies could target general cognition instead of focusing on specific cognitive domains and provide targeted memory strategies focusing on encoding and retrieval abilities. Additionally, the intervention should include systems designed to sustain treatment gains, such as booster sessions, activity monitoring, and feedback mechanisms, which may address limitations observed in the current intervention’s efficacy. These findings offer valuable guidance for the design of future trials for post-stroke cognitive rehabilitation.

### 4.1 Limitations

Firstly, the results are not generalizable to non-English speaking participants as this pilot trial was limited to English-speaking participants. This is relevant in a multilingual and multicultural society such as Singapore. Future studies can be replicated in different languages. Secondly, this study comprised of a small sample size that did not include randomization or a control group, hence reducing the power of the statistical analysis in detecting effects from the intervention. Thirdly, adjustments for confounding variables were not made due to the pilot nature of the study and small sample size. Fourthly, as mentioned before, there was no control over time elapsed post-stroke as participants at various stages of stroke recovery were included, which would impact upon the effects of the intervention on different individuals. Future studies should control for participants’ stage of recovery after stroke.

### 4.2 Conclusion

Overall, our findings suggest that while the TYB program show promise in leading to some short-term stabilization in cognitive functioning among stroke survivors, these gains may not be sustained over time. The persistent cognitive deficits observed among stroke survivors underscore the need for future research to focus on developing comprehensive interventions aimed at addressing the complex cognitive challenges faced by stroke survivors, with a particular emphasis on long-term maintenance of cognitive functioning. Future larger-scale studies should also take into consideration the limitations of this pilot study.

## Data Availability

The raw data supporting the conclusions of this article will be made available by the authors without undue reservation.
