# Supplementary figures and images for: Train-your-brain pilot community-based intervention after stroke: cognitive trajectory over 10-month follow-up
Source: Front Neurol. 2025 Jul 7;16:1500943. doi: 10.3389/fneur.2025.1500943 (PMC12277129; doi:10.3389/fneur.2025.1500943)

Supplementary Material


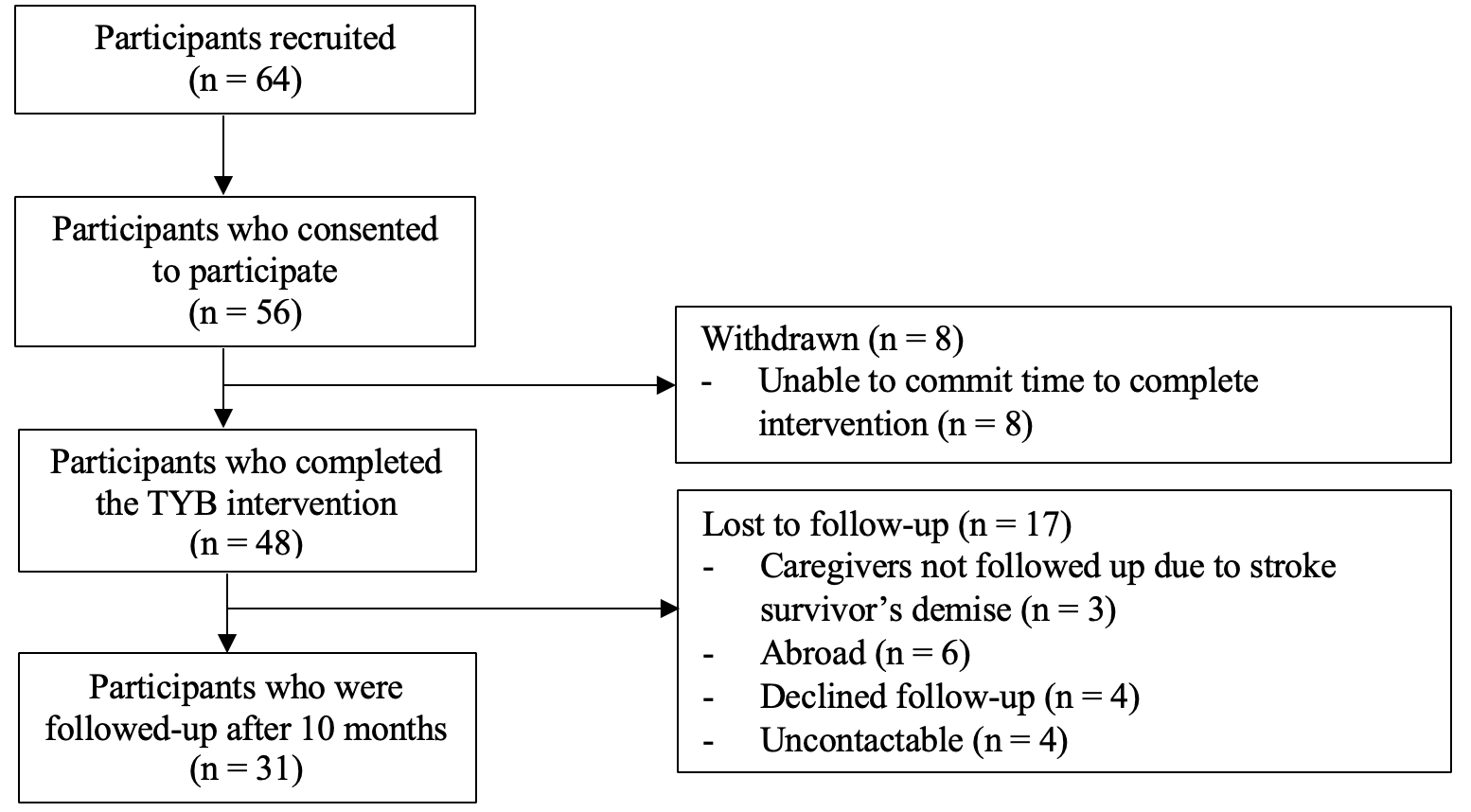


**Fig. 1** Flow diagram of participation during the study

Supplement: Supplementary file 1 [file Table_1.docx]
